# Management of hyperkalemia during treatment with mineralocorticoid receptor blockers: findings from esaxerenone

**DOI:** 10.1038/s41440-020-00569-y

**Published:** 2020-11-20

**Authors:** Hiromi Rakugi, Satoru Yamakawa, Kotaro Sugimoto

**Affiliations:** 1 Department of Geriatric and General Medicine, Osaka University Graduate School of Medicine, 2-2 Yamadaoka, Suita, Osaka 565-0871 Japan; 2 Clinical Development Department III, R&D Division, Daiichi Sankyo Co., Ltd., 1-2-58, Hiromachi, Shinagawa-ku, Tokyo 140-8710 Japan; 3 Medical Science Department, Daiichi Sankyo Co., Ltd., 3-5-1, Nihonbashi Honcho, Chuo-ku, Tokyo 103-8426 Japan

**Keywords:** Esaxerenone, Mineralocorticoid receptor blocker, Hypertension, Hyperkalemia management

## Abstract

The nonsteroidal mineralocorticoid receptor (MR) blocker esaxerenone has demonstrated good antihypertensive activity in a variety of patients, including those with uncomplicated grade I–III hypertension, hypertension with moderate renal dysfunction, hypertension with type 2 diabetes mellitus with albuminuria, and hypertension associated with primary aldosteronism. Hyperkalemia has long been recognized as a potential side effect occurring during treatment with MR blockers, but there is a lack of understanding and guidance about the appropriate management of hyperkalemia during antihypertensive therapy with MR blockers, especially in regard to the newer agent esaxerenone. In this article, we first highlight risk factors for hyperkalemia, including advanced chronic kidney disease, diabetes mellitus, cardiovascular disease, age, and use of renin-angiotensin-aldosterone system inhibitors. Next, we examine approaches to prevention and management, including potassium monitoring, diet, and the use of appropriate therapeutic techniques. Finally, we summarize the currently available data for esaxerenone and hyperkalemia. Proper management of serum potassium is required to ensure safe clinical use of MR blockers, including awareness of at-risk patient groups, choosing appropriate dosages for therapy initiation and dosage titration, and monitoring of serum potassium during therapy. It is critical that physicians take such factors into consideration to optimize MR blocker therapy in patients with hypertension.

## Introduction

Mineralocorticoid receptor (MR) blockers are a class of drugs used in the treatment of essential hypertension and hyperaldosteronism and have antihypertensive effects in patients with low-renin or refractory (resistant) hypertension; these effects improve prognosis in patients with heart failure [1–9]. A recent meta-analysis of data from 3.2 million patients indicated a high prevalence and worldwide burden of resistant hypertension and a need for new and more effective treatments [10]. The first MR blocker identified was spironolactone, a nonselective agent that also had affinity for progesterone and androgen receptors; this lack of selectivity and non-MR blocking activity contributed to the occurrence of problematic adverse events, including menstrual abnormalities in women and sexual dysfunction with painful gynecomastia in men [11–13]. Eplerenone, a second-generation MR blocker, had improved specificity for the MR, thus reducing the frequency of serious sex hormone-related adverse events [14–16]. More recently, the highly selective nonsteroidal MR blockers esaxerenone, finerenone, apararenone, and AZD9977 have been successively developed [17–21].

Among the nonsteroidal MR blockers, esaxerenone (MINNEBRO^®^, Daiichi Sankyo Co., Ltd.) was approved in Japan in January 2019 for the treatment of hypertension [22, 23]. In vitro studies showed that esaxerenone specifically inhibits the binding of aldosterone to MR and does not have agonistic or antagonistic effects on glucocorticoid, androgen, or progesterone receptors, even at high concentrations [24]. Owing to the high MR specificity of esaxerenone, the incidence of sex hormone-related adverse events in phase III studies was very low (overall safety assessment: 2/1,250 [0.16%]; Daiichi Sankyo Co., Ltd., unpublished data).

Esaxerenone has demonstrated good antihypertensive activity in a variety of patients, including those with uncomplicated grade I–III hypertension, hypertension with moderate renal impairment, hypertension with type 2 diabetes mellitus with albuminuria, and hypertension associated with primary aldosteronism (Daiichi Sankyo Co., Ltd., unpublished data, J305 and J307 studies; and published data [25–28]). The currently available clinical study data are summarized in Table 1. Esaxerenone 2.5 mg/day has been shown to be noninferior to eplerenone 50 mg/day with respect to antihypertensive effects [25]. The antihypertensive effects of esaxerenone persisted throughout 52 weeks of treatment even when used as a monotherapy or when it was administered as an add-on therapy with renin-angiotensin system (RAS) inhibitors or calcium channel blockers (CCBs) [27]. In addition to its antihypertensive effects in hypertensive patients with type 2 diabetes and albuminuria, renoprotective effects have also been reported, with the urinary albumin/creatinine ratio decreasing by 32.4% from baseline during treatment with 5 mg/day esaxerenone, even in the presence of RAS inhibitors [26]. Similar antihypertensive and renoprotective effects have been observed in patients with moderate kidney dysfunction (Daiichi Sankyo Co., Ltd., unpublished data, J305 study). In contrast, eplerenone is contraindicated for hypertensive patients with moderate renal impairment and those with type 2 diabetes mellitus with albuminuria [16].

**Table 1:** Blood pressure-lowering effects of esaxerenone in phase III clinical studies (changes in sitting blood pressure)

Study name	Patients	Other antihypertensive drugs	Esaxerenone dose	SBP/DBP, mmHg	Eplerenone 50 mg SBP/DBP, mmHg
Comparison study (ESAX-HTN: J301 [25])	Grade I or II essential hypertension	None	2.5 mg^a^ 5 mg^a^	−13.7/−6.8^c^ (*n* = 306) −16.9/−8.4^c^ (*n* = 322)	−12.1/−6.1^c^ (*n* = 316)
Long-term administration (J302 [27])	Grade I or II essential hypertension	None, CCB, or RAS inhibitor	2.5–5 mg^b^	12 weeks: −16.1/−7.7 (*n* = 368) 28 weeks: −18.9/−9.9 (*n* = 368) 52 weeks: −23.1/−12.5 (*n* = 147)	—
J304 [28]	Grade III hypertension	None, RAS inhibitor	2.5–5 mg^b^	−21.0/−13.2 (*n* = 20)	—
J305 (unpublished data)	Hypertension with moderate renal impairment	RAS inhibitor	1.25–5 mg^b^	−17.8/−8.1 (*n* = 58)	— ^d^
J306 [26]	Hypertension with type 2 diabetes with albuminuria	RAS inhibitor	1.25–5 mg^b^	−13.7/−6.2 (*n* = 51)	— ^d^
J307 (unpublished data)	Primary aldosteronism	None, CCB	2.5–5 mg^b^	−17.7/−9.5 (*n* = 44)	—

*CCB* calcium channel blocker, *RAS* renin-angiotensin system

^a^Non-approved administration regimen (fixed dose)

^b^The esaxerenone dosage was gradually increased

^c^Per protocol set

^d^Contraindication

Thus, MR blockers have an important role in the management of hypertension, particularly in patients who cannot achieve adequate blood pressure reduction. Hyperkalemia is, however, recognized as a potential side effect during treatment with MR blockers [14, 15, 29, 30]. Publication of data from the Randomized Aldactone Evaluation Study showed that spironolactone reduced mortality by 30% compared with the mortality rate associated with placebo in patients with severe heart failure already receiving angiotensin-converting enzyme (ACE) inhibitors [6]. As a consequence, a marked increase in the prescription of spironolactone was observed in Canada [31]; however, this increase was associated with an increase in hyperkalemia-related hospitalizations and deaths [31]. Regarding esaxerenone, during the phase III clinical studies conducted in Japan, blood potassium increased was the most common side effect (occurring in 4.1% of patients) [32]. As such, concerns about the occurrence of hyperkalemia during therapy are growing, but there is a lack of understanding and guidance about the appropriate management of hyperkalemia during antihypertensive therapy with MR blockers, especially with the newer agent esaxerenone. Therefore, this article highlights risk factors for hyperkalemia and approaches for its prevention and management and summarizes currently available data for esaxerenone.

## Definitions of hyperkalemia

Definitions of hyperkalemia vary by geographic region. In Europe, mild hyperkalemia is defined as serum potassium 5.0–5.4 mEq/L, moderate as 5.5–5.9 mEq/L, and severe as ≥6.0 mEq/L [33]. The American Heart Association uses cutoffs of 5–6 mEq/L (mild), 6–7 mEq/L (moderate), and >7 mEq/L (severe) [34]. However, in this review, we refer to the standard potassium values specified in the guidelines published by the Japanese Ministry of Health, Labor and Welfare entitled “Classification criteria for the seriousness of adverse drug reactions of medical agents”, wherein serum potassium values of ≥5.5 mEq/L and <6.0 mEq/L are classified as Grade 2 adverse drug reactions (ADRs), and values of ≥6.0 mEq/L are classified as Grade 3 ADRs [35]. For patients with chronic kidney disease (CKD), Japanese nephrology guidelines recommend maintaining serum potassium between 4.0 and 5.5 mEq/L to avoid hypo- or hyperkalemia [36]. Of note, mild to moderate hyperkalemia generally cannot be diagnosed from electrocardiogram (ECG) changes; a study in hospitalized patients indicated that the first ECG changes occurred at serum potassium levels above 7.2 mEq/L [37], a level that already indicates severe hyperkalemia [33, 34].

## Hyperkalemia risk factors and prognosis

### Prognosis

Potassium homeostasis regulates antiarrhythmic effects, maintenance of diastolic function, vasodilation, and reduction of thrombosis and atherosclerosis [38]. Thus, disturbed potassium homeostasis is associated with a number of adverse events. High potassium levels are associated with myocardial infarction, cardiac hyperexcitability and depression, neuromuscular manifestations (including paresthesia, muscle cramps, and muscle weakness), and gastrointestinal symptoms (including nausea and vomiting) [39–44]. Low potassium levels are also associated with muscle weakness and fatigue, as well as neurologic symptoms (including confusion and affective disorders), smooth muscle dysfunction (including paralytic ileus), arrhythmias and myocardial infarction, and even glucose intolerance [41, 43–47]. Collectively, the association between serum potassium levels and mortality shows a U-shaped relationship, with increased mortality observed when serum potassium is either high or low [48]. In one analysis in patients with CKD (estimated glomerular filtration rate (eGFR) <60 mL/min/1.73 m^2^), the pooled adjusted incidence rate ratios for patients with potassium levels <3.5 and ≥6.0 mEq/L were 3.05 (95% confidence interval (CI) 2.53–3.68) and 3.31 (95% CI 2.52–4.34), respectively [49].

Many studies have shown that very high serum potassium levels predispose patients to sudden death [50]. More recently, Kashihara et al. reported that in Japanese patients with CKD Stage 3a with serum potassium ≥6.0 mEq/L, the 3-year mortality rate was 22.64% [51]. Hypokalemia (serum potassium <4.0 mEq/L) has also been reported to correlate with worse prognosis and increased mortality in patients with heart disease, with or without CKD [52, 53]. Specifically, in chronic heart failure patients, hypokalemia increases the risk of death [54]. Furthermore, compared with normokalemia, serum potassium levels <3.5 mEq/L have been associated with an increased risk of developing atrial fibrillation (hazard ratio (HR) 1.63, 95% CI 1.03–3.56) [55], while levels <3.0 mEq/L induced a number of proarrhythmic changes, contributing to the occurrence of fatal ventricular arrhythmias and sudden cardiac death [56]. Therefore, clinicians need to be aware of the dangers of both hyperkalemia and hypokalemia.

### Risk factors

Advanced CKD, diabetes mellitus, and cardiovascular disease (CVD) have all been identified as independent predictors of hyperkalemia [57]. In the real world, dehydration and diet may also be triggers for hyperkalemia [58]; dehydration is less likely in a clinical trial situation where patients are extensively screened for medical issues prior to inclusion. Finally, in general clinical practice, one of the most common risk factors for hyperkalemia is pharmacologic medication, including renin-angiotensin-aldosterone system inhibitors (RAASis) [59].

In a recently published Japanese study of hospital claims data from >1 million Japanese patients, 6.8% were found to have hyperkalemia [51]; this prevalence was found to be higher in patients with CKD (22.8%), heart failure (13.4%), diabetes (10.8%), and hypertension (10.8%). Furthermore, in patients receiving RAASi treatment, the incidence and prevalence of hyperkalemia (14.2%) was higher than that in the general population [51].

#### CKD (low eGFR)

The kidney is usually the main route of potassium excretion; thus, in patients with CKD, urinary potassium excretion declines as the GFR decreases [60]. The frequency of hyperkalemia correlates with the level of renal function; in a multivariate analysis of elderly patients (aged ≥ 65 years) with CKD and an eGFR <60 mL/min/1.73 m^2^, the odds ratio (OR) for hyperkalemia increased by 1.26 for every 5 mL/min/1.73 m^2^ decrease in eGFR [61]. Similarly, data from 1,094 African American adults with hypertensive CKD showed an increased risk of hyperkalemia in patients with an eGFR of <30 or 31–40 mL/min/1.73 m^2^ versus those with an eGFR of >50 mL/min/1.73 m^2^ (HR 3.61, 95% CI 1.42–9.18; *p* = 0.007) [62]. Furthermore, data from a retrospective cohort analysis indicated that during antihypertensive treatment with the MR blocker spironolactone, patients with CKD had a significantly higher rate of hyperkalemia (>5.5 mEq/L) than those without CKD (5.7% vs. 0%) [63].

#### Diabetes

There are a number of mechanisms that contribute to a higher risk of developing hyperkalemia in patients with diabetes; these include impaired potassium excretion, impaired renal tubular function, and a reduced ability to shift potassium into cells [64]. Diabetes was identified as an independent predictor of hyperkalemia in a large retrospective cohort study conducted in the United States [65]. In a nested case–control study of CKD patients with or without diabetes, the presence of diabetes significantly increased the prevalence of hyperkalemia in patients with Stage 3 CKD (28.6% vs. 17.5% in those without diabetes; *p* = 0.036) [66]. In a recent registry study in Denmark, one in six patients with newly diagnosed diabetes experienced a hyperkalemic event within 4 years, and the development of hyperkalemia in patients with diabetes was associated with worse clinical outcomes and higher mortality rates [67].

#### CVD

Patients with heart failure are often older [68] and have a high prevalence of CKD [69], both of which are risk factors for the development of hyperkalemia [51, 70, 71]. Thus, in patients with heart failure, abnormalities in electrolyte levels, resulting from both the pathophysiologic changes underlying the disease and from the therapeutic regimens used in treatment, are a common and potentially hazardous complication [72, 73].

Based on data from a large retrospective cohort study of patients with CVD and CKD (*n* = 15,803), both coronary artery disease (HR 1.32, 95% CI 1.21–1.43) and peripheral vascular disease (HR 1.55, 95% CI 1.36–1.77) have been found to be predictors of hyperkalemia [57]. In a cohort of 19,194 patients with newly diagnosed heart failure followed for a mean of 3.9 years, 2,176 cases of hyperkalemia were identified (11.3%) [74]. Significant risk factors included valvular heart disease (OR 1.28; 95% CI 1.06–1.54), renal failure (OR 3.81; 95% CI 3.29–4 .42), and diabetes (OR 1.52; 95% CI 1.31–1.75), as well as the use of pharmacologic medication (including potassium-sparing diuretics (OR 3.01; 95% CI 2.61–3.48) and ACE inhibitors (OR 1.70; 95% CI 1.41–2.04)) [74].

#### Age

Distal renal tubule function and the ability to eliminate potassium decline as age increases [75–77]. As a result, elderly individuals are at increased risk of developing hyperkalemia, especially those who also have CKD or CVD [78]. In a group of patients with heart failure, the mean (±standard deviation) age was significantly older in those with serum potassium >5.0 mEq/L (76 ± 9 years) than in those with lower potassium levels (73 ± 11 years) [79]. Similarly, in a retrospective study of patients with CVD and CKD, patients with hyperkalemia were significantly older (66.1 ± 10.3 years) than those with normal potassium levels (63.9 ± 11.2 years) [57]. The results of another retrospective analysis using electronic medical records showed that older patients were at greater risk of developing hyperkalemia (serum potassium >5.0 mEq/L) than younger patients (OR (95% CI) values for age 45–64, 65–74, and ≥75 years versus age 18–44 years were 1.41 (1.36–1.47), 1.66 (1.59–1.73) and 1.72 (1.65–1.79), respectively) [65].

#### RAASi medication

Treatment with antihypertensive agents, ACE inhibitors, angiotensin receptor blockers (ARBs), a direct renin inhibitor, and MR blockers are all known to increase the risk of hyperkalemia [59, 80, 81]. Elevated serum potassium levels are observed with single-agent administration, and the level increases further with combination therapy [82, 83]. A systematic review showed that a combination therapy with an ACE inhibitor and an ARB resulted in a small, but significant, increase in serum potassium levels (weighted mean difference, 0.11 mEq/L; 95% CI 0.05–0.17) [84].

In a study of normokalemic patients with hypertension who were randomly assigned to treatment with the ACE inhibitor lisinopril, the diuretic chlorthalidone, or the CCB amlodipine, the incidence of hyperkalemia was found to be greater in the patients treated with lisinopril (3.6%) than in those treated with either of the other agents (*p* < 0.01 for both) [85]. In another study of patients with systemic hypertension, monotherapy with either the ACE inhibitor enalapril or the MR blocker eplerenone was associated with elevated serum potassium levels [86].

In patients with diabetes, multiple studies have reported that treatment with MR blockers [87, 88], ARBs [89, 90], ACE inhibitors [90], or a direct renin inhibitor [91] is associated with an increase in potassium levels and a risk of severe hyperkalemia in a small number of patients. In patients with systolic heart failure, large randomized studies investigating ACE inhibitors or ARBs have demonstrated significant increases in serum potassium during treatment. In one study, the mean serum potassium level increased by 0.2 mEq/L during treatment with enalapril, and the proportion of patients with a serum potassium level >5.5 mEq/L was significantly higher in the enalapril group than in the placebo group (6.4% vs. 2.5%; *p* < 0.01) [92]. Similarly, the rate of hyperkalemia leading to treatment discontinuation was higher in the candesartan group than in the placebo group in a large randomized controlled trial (RCT) (1.9% vs. 0.3%; *p* = 0.0005) [93].

Combination therapy has the potential to further worsen the risk of hyperkalemia. In one study, the addition of spironolactone or losartan to ACE inhibitor therapy in patients with hypertension, diabetes, and albuminuria was associated with significant increases in serum potassium level compared to the outcome in the placebo group (*p* < 0.0001 and *p* = 0.03, respectively) [94]. In parallel-group RCTs of spironolactone added to an ACE inhibitor and/or ARB, increases in serum potassium ranged between 0.1 and 0.8 mEq/L [6, 88, 94–97]. Corresponding increases in serum potassium in crossover RCTs were 0.1–0.3 mEq/L [87, 98–100], and in nonrandomized trials, they were 0.1–0.4 mEq/L [101–104].

### Management of hyperkalemia in hypertension

To ensure patient safety, it is important to monitor and manage potassium at a personalized level owing to interindividual variations in risk, symptoms, and treatments.

#### Monitoring

In the general patient population, potassium levels are often elevated. Approximately 6.8% of the Japanese population is estimated to have hyperkalemia (defined as at least two serum potassium readings ≥5.1 mEq/L) based on the results of a medical database analysis [51]. Similarly, a Swedish study examining 364,955 patients who accessed healthcare over a 3-year period revealed a 7% incidence of hyperkalemia (defined as potassium >5 mEq/L) with a 35.7% rate of recurrence [105].

Regular monitoring allows drug dosages to be adjusted based on serum potassium levels. A long-term potassium monitoring study in patients with heart failure suggested that maintenance of serum potassium levels within the normal range should be considered a therapeutic target, allowing clinicians to modify treatment and mitigate risks to improve clinical outcomes for patients [79]. In patients with diabetes being treated with an ACE inhibitor, ARB, or spironolactone, compared with no monitoring, regular monitoring of serum potassium has been shown to decrease the risk of hyperkalemia-related adverse events (adjusted relative risk 0.5, 95% CI 0.37–0.66) [64]. According to the Kidney Disease Outcomes Quality Initiative [106], the suggested frequency of serum potassium monitoring after initiation of ACE inhibitor or ARB therapy is as follows: every 4–12 weeks for serum potassium ≤4.5 mEq/L, every 2–4 weeks for 4.6–5 mEq/L, and more frequently than every 2 weeks for >5 mEq/L. The American Heart Association states that potassium levels and renal function should typically be checked on days 3 and 7 after initiating therapy with aldosterone antagonists and then at least monthly for the first 3 months [107]. However, the frequency of serum potassium measurement is not described in the guidelines published by the Japanese Society of Nephrology [36] or Japanese societies related to CVD [108].

To avoid the risk of false positive findings, serum potassium levels need to be measured carefully [109]. Causes of false positives include hemolysis, delay in sample processing, long-term cold storage, and contamination of blood samples with antiseptics or potassium salts from blood collection tubes [110, 111]. Careful collection techniques and proper sample handling and storage are therefore essential.

Additional suggestions for clinicians intending to prescribe RAASis include pretreatment GFR and baseline serum potassium determination (to identify or exclude high-risk patients), dosage titration, and discontinuation of potassium supplements [107] or concomitant maintenance treatment with loop or thiazide diuretics [112].

#### Diet

Restricting the intake of potassium-rich foods is also effective in preventing hyperkalemia, and restricting dietary potassium intake is particularly important for patients with renal impairment [113]. According to Japanese dietary recommendations for CKD [114], although there is no specific limit for patients with CKD Stages 1–3a (eGFR ≥ 45 mL/min/1.73 m^2^), daily potassium intake should be limited to ≤2,000 mg/day for patients with Stage 3b (eGFR 30 to <45 mL/min/1.73 m^2^) and to ≤1,500 mg/day for patients with Stages 4 or 5 (eGFR < 30 mL/min/1.73 m^2^). In adults with Stages 3–5d and posttransplant patients with a normal potassium range, the National Kidney Foundation suggests that it is reasonable to adjust the dietary potassium intake to maintain levels within the normal range, but if this population exhibits hyperkalemia, their dietary potassium level should be reduced for the adjustment of serum potassium levels [115]. Clinicians should, therefore, be aware of foods that are high or low in potassium. For example, the potassium content of syrup, vegetable oils, and shortenings is zero [116], while that of dairy products, fruits such as bananas and pineapples, and vegetables such as pumpkins and sweet potatoes is particularly high [113]. As a result, patients who are planning to adjust their potassium intake must limit the amount of these foods consumed. Conversely, a diet in which various food groups are extremely restricted will result in a lack of essential nutrients and may cause nutritional disorders [117]. In addition, even for the same food item, the amount of potassium consumed can vary depending on the cooking method utilized [113]. In the event that potassium limitation is deemed necessary, appropriate guidance should be provided in coordination with a registered dietician.

#### Treatment

Approaches to managing hyperkalemia depend upon symptom severity and include dietary potassium intake restriction, dosage adjustment of MR blockers and/or RAS inhibitors, promotion of potassium excretion from the body using diuretics, use of oral potassium adsorbent agents, such as calcium/sodium polystyrene sulfonate, promotion of potassium redistribution from extracellular to intracellular spaces via intravenous administration of insulin and glucose, cell membrane stabilization via intravenous administration of calcium solution, and hemodialysis [118–120].

For patients with mild hyperkalemia (<6.0 mEq/L) without ECG changes, a reduced potassium intake and discontinuation of potassium-elevating drugs may be sufficient to normalize potassium levels [118]. Strategies to remove potassium from the body, such as the use of loop diuretics and oral potassium adsorbent agents, should be considered for emergent or symptomatic hyperkalemia [58]. Administration of sodium polystyrene sulfonate causes gastrointestinal excretion of potassium, but careful monitoring is necessary as the rate of excretion may be unpredictable [118].

These same procedures are generally also effective for severe hyperkalemia (>6.5 mEq/L) without ECG changes. If more aggressive therapy is required, potassium can be redistributed using insulin and glucose, with a peak occurring 60 min after administration and continuing for several hours [118]; however, these agents may be contraindicated in patients with certain comorbidities. During insulin administration, glucose is administered simultaneously to prevent hypoglycemia caused by increased uptake of glucose alongside potassium. For severe hyperkalemia (>6.5 mEq/L), particularly in patients with cardiomyocyte damage in whom an ECG trace shows the loss of P-wave or QRS widening, treatment with intravenous calcium to antagonize the action of potassium in the heart may also be indicated [118]. In emergency cases, hemodialysis is necessary.

### Esaxerenone and hyperkalemia

A summary of the incidence of serum potassium elevation occurring in phase III clinical trials of esaxerenone is provided in Table 2. These trials included patients with essential hypertension, hypertension with moderate renal impairment, hypertension with type 2 diabetes and albuminuria, and primary aldosteronism, and esaxerenone was given alone or in combination with RAS inhibitors or CCBs (Daiichi Sankyo Co., Ltd., unpublished data, J305, and J307 studies; and published data [25–28]). In these clinical studies, serum potassium elevation was defined as a serum potassium level ≥5.5 mEq/L; this level was set to assure patient safety and to reduce the risk of patients developing dangerously high potassium levels of ≥6.0 mEq/L.

**Table 2:** Incidence of serum potassium elevation in the esaxerenone phase III clinical trials^a^

Study name		Esaxerenone dose	Age (y) at baseline, mean ± SD	eGFR (mL/min/1.73 m^2^) at baseline, mean ± SD	Serum potassium (mEq/L) at baseline, mean ± SD	RAS inhibitor	Total *N*	Frequency of serum potassium elevation	
								≥5.5 mEq/L, *n* (%)	≥6.0 or ≥5.5 mEq/L on two consecutive occasions, *n* (%)
Grade I or II essential hypertension, comparison study (J301 [25])		Fixed dose^b^ 2.5 mg	55.9 ± 9.2^c^	78.3 ± 12.4^c^	4.19 ± 0.28^c^	None	330^c^/331^d^	15 (4.5)^d^	3 (0.9)^d^
		Fixed dose^b^ 5 mg	54.8 ± 9.7^c^	79.3 ± 12.4^c^	4.22 ± 0.30^c^	None	337^c^/338^d^	10 (3.0)^d^	2 (0.6)^d^
Grade I or II essential hypertension, long-term administration (J302 [27])	Esaxerenone alone	Incremental dose 2.5–5 mg	55.9 ± 9.4	79.2 ± 13.1	4.18 ± 0.27	None	245	14 (5.7)	4 (1.6)
	Combination with CCB	Incremental dose 2.5–5 mg	56.1 ± 8.9	82.7 ± 13.3	4.15 ± 0.26	None	59	2 (3.4)	0 (0.0)
	Combination with RAS inhibitor	Incremental dose 2.5–5 mg	57.2 ± 8.9	78.4 ± 10.0	4.16 ± 0.29	Yes	64	4 (6.3)	0 (0.0)
Grade III hypertension (J304 [28])		Incremental dose 2.5–5 mg	52.7 ± 10.0	78.8 ± 13.5	4.17 ± 0.28	None (85%) Yes (15%)	20	0 (0.0)	0 (0.0)
Hypertension with moderate renal impairment (J305, unpublished data)		Incremental dose 1.25–5 mg	68.0 ± 7.7	50.9 ± 6.5	4.29 ± 0.27	Yes	58	7 (12.1)	0 (0.0)
Hypertension with type 2 diabetes with albuminuria (J306 [26])		Incremental dose 1.25–5 mg	63.0 ± 9.8	73.1 ± 19.5	4.20 ± 0.28	Yes	51	2 (3.9)	1 (2.0)
Primary aldosteronism (J307, unpublished data)		Incremental dose 2.5–5 mg	49.6 ± 9.7	78.5 ± 13.8	4.01 ± 0.33	None	44	1 (2.3)	1 (2.3)

*CCB* calcium channel blocker, *eGFR* estimated glomerular filtration rate, *RAS* renin-angiotensin system, *SD* standard deviation

^a^Includes all patients with serum potassium elevation, whether or not elevated potassium was reported as a side effect

^b^Non-approved administration regimen

^c^Full analysis set

^d^Safety analysis set

Overall, in the phase III studies, the incidence of blood potassium increased as a side effect was 4.1% (51/1,250 patients), and of these patients, 1.7% (21/1,250 patients) had serum potassium levels ≥5.5 mEq/L [32]. There were no notable differences between the incidence rates of serum potassium elevation observed with esaxerenone and those reported for eplerenone and spironolactone. In patients with hypertension without severe renal impairment or diabetes who received monotherapy, increases in potassium levels ≥5.5 mEq/L occurred in 3.0–5.7% of esaxerenone-treated patients [25, 27], compared with 1.8–3.0% of eplerenone-treated patients [25, 121]. In this same patient population, when administered in combination with a RAS inhibitor or CCB, potassium levels ≥5.5 mEq/L occurred in 3.4–6.3% [27] and 0–1.2% [122] of esaxerenone- and eplerenone-treated patients, respectively. In patients with diabetes and albuminuria, potassium levels ≥5.5 mEq/L occurred in 3.9% of esaxerenone-treated patients [26] and in 4.8% of spironolactone-treated patients [98]. Although incidence rates of serum potassium elevation may appear to be slightly numerically higher for esaxerenone than for eplerenone, only a single phase III study in patients with essential hypertension and normal kidney function (J301 [25]) has conducted a direct comparison of esaxerenone and eplerenone within the same clinical trial, and no statistical analysis was conducted in regard to the rates of serum potassium elevation between esaxerenone and eplerenone in the study. Moreover, no comparator study exists for patients with severe renal impairment or diabetes. Thus, regarding the incidence of hyperkalemia, the superiority or inferiority of either of these agents over the other remains unproven.

**Fig. 1:**
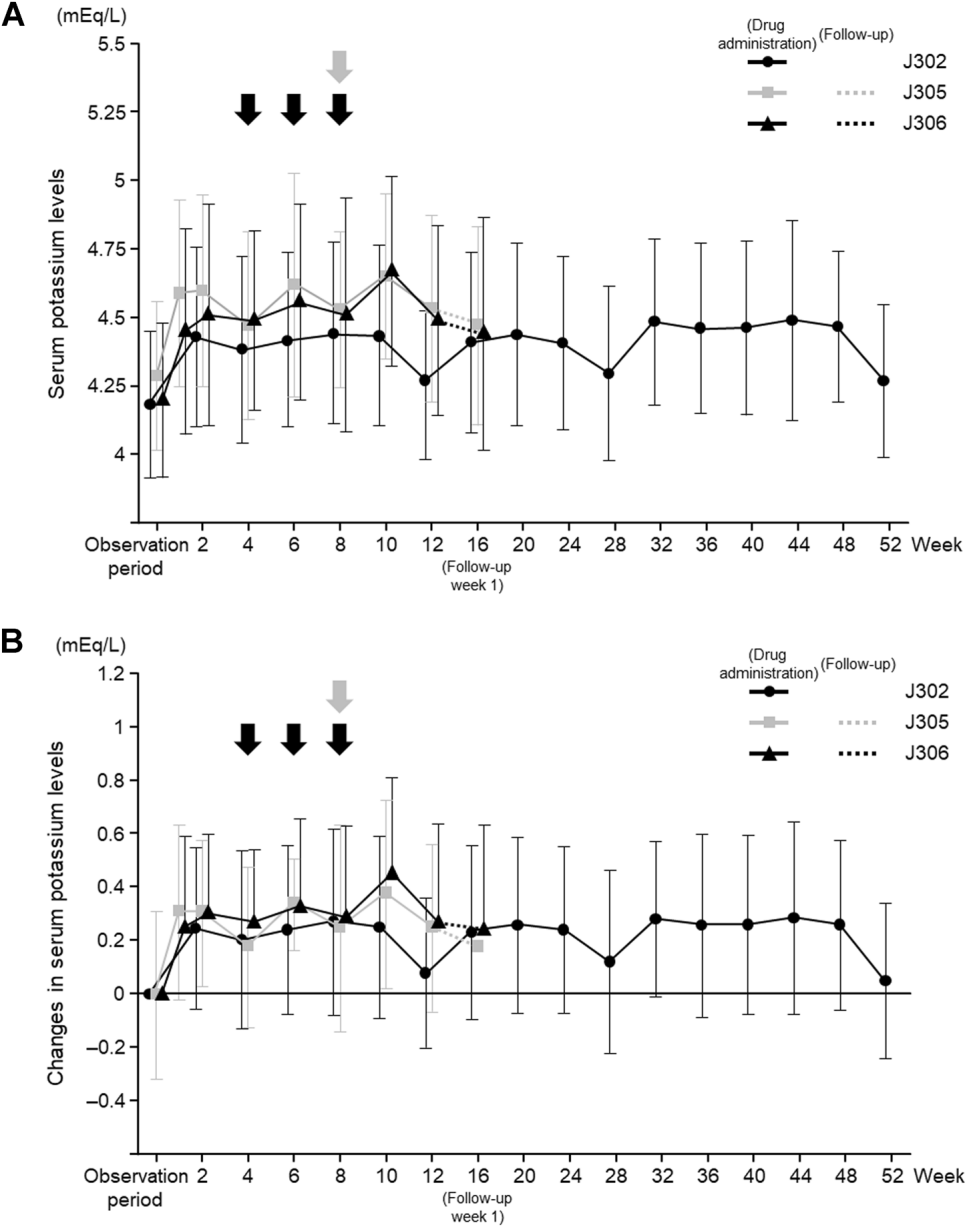
Serum potassium levels over time according to treatment regimen. **A** Changes in serum potassium levels. **B** Changes in serum potassium from baseline. Black arrows indicate dose increases at weeks 4, 6, and 8: 2.5–5 mg for the J302 [27] study and 1.25–2.5 mg for the J305 (unpublished data) and J306 [26] studies. The gray arrow indicates dose increases at week 8: 2.5–5 mg for the J305 (unpublished data) and J306 [26] studies. Solid lines indicate dose periods, and dotted lines indicate follow-up (no treatment) periods

Data for the time course of serum potassium levels during esaxerenone treatment are shown in Fig. 1. These study data indicate that increases in serum potassium levels occurred in the first 2 weeks after treatment initiation, without any additional increases at the time of esaxerenone dose escalation [27]. There was no specific trend in the onset time of serum potassium elevation after 2 weeks of esaxerenone administration. Based on these data, the esaxerenone labeling recommendations suggest that potassium levels should be measured before treatment, at 2 and 4 weeks after treatment initiation (or during any up- or down-titration), and regularly thereafter [22]. Similarly, studies of eplerenone in patients with heart failure demonstrated that elevated potassium levels were detectable within the first 1–4 weeks after treatment initiation [123, 124], and a study of spironolactone in patients with CKD also indicated that serum potassium increased within the first 4 weeks of treatment [125].

The profile of serum potassium levels following esaxerenone treatment remained consistent in high-risk patient groups (i.e., those with moderate renal dysfunction or diabetes with albuminuria) (Fig. 1) (Daiichi Sankyo Co., Ltd., unpublished data, J305 study; and published data, J306 study [26]). In patients with moderate renal impairment (J305) and type 2 diabetes and albuminuria (J306) [26], mean serum potassium levels increased following the initiation of esaxerenone treatment and after each dose escalation visit, but there was no clear trend towards a continuous increase over time. In the long-term administration study (J302), there was no association between duration of treatment and incidence of serum potassium elevation. The detailed timing of onset of serum potassium elevation is shown in Supplementary Table 1 (moderate renal impairment; unpublished data, J305), Supplementary Table 2 (type 2 diabetes and albuminuria [126]), and Supplementary Table 3 (long-term administration study [27]).

There were no sex-related differences in the incidence of serum potassium elevation in esaxerenone-treated patients. There were also no differences in rates of serum potassium elevation during esaxerenone therapy according to body weight, liver function, or use of a RAS inhibitor (even though, as described earlier, RAS inhibitors alone have been shown to increase the risk of elevated serum potassium level) (Supplementary Table 4). Regarding liver function, mild to moderate hepatic impairment was shown to have no clinically relevant effect on esaxerenone exposure [127]. Conversely, increased blood exposure of eplerenone was observed in patients with mild to moderate hepatic impairment, but this increase was not associated with the incidence of hyperkalemia [16, 128]. Thus, the liver function does not appear to be associated with serum potassium elevation.

Table 3 shows rates of serum potassium elevation in patient subgroups based on baseline serum potassium, baseline renal function, age, and presence of type 2 diabetes with albuminuria. The proportion of patients with serum potassium ≥5.5 mEq/L was higher among patients with baseline potassium levels ≥4.5 than among those with baseline potassium levels <4.5 mEq/L and in the subgroup with eGFR <60 mL/min/1.73 m^2^ than in the subgroup with eGFR ≥60 mL/min/1.73 m^2^ (Table 3). Thus, it appears that serum potassium levels are more likely to be elevated if renal function is worse. However, neither baseline serum potassium nor eGFR had any effect on the proportion of patients with serum potassium ≥6.0 or 5.5 mEq/L on two or more consecutive occasions. There was no clear trend in the occurrence of elevated potassium levels according to age (<65, ≥65, or ≥75 years) in esaxerenone-treated patients (Table 3). Nevertheless, because the renal function is known to decline with age, thorough monitoring is recommended in elderly patients. No tendency towards serum potassium elevation was observed in patients with type 2 diabetes and albuminuria (Table 3).

**Table 3:** Patients at risk for serum potassium elevation^a^

Stratified by baseline serum potassium					
Study name	Esaxerenone dose	Baseline serum potassium (mEq/L)	*N*	Serum potassium ≥5.5 mEq/L, *n* (%)	Serum potassium ≥6.0 or ≥5.5 mEq/L on two consecutive occasions *n* (%)
J203 [131]/J301 [25]	Fixed dose^b^	<4.5	764	9 (1.2)	1 (0.1)
	1.25–5 mg	≥4.5	160	21 (13.1)	5 (3.1)
J302 [27]	Incremental dose	<4.5	310	11 (3.5)	2 (0.6)
	2.5–5 mg^c^	≥4.5	58	9 (15.5)	2 (3.4)

Stratified by renal function					
Study name	Esaxerenone dose	Baseline eGFR (mL/min/1.73 m^2^)	*N*	Serum potassium ≥5.5 mEq/L, *n* (%)	Serum potassiu ≥6.0 or ≥5.5 mEq/L on two consecutive occasions, *n* (%)
J204 [126]	Fixed dose^b^	≥30, <60	100	21 (21.0)	7 (7.0)
	0.625–5 mg^c^	≥60	175	13 (7.4)	5 (2.9)
J306 [26]	Incremental dose	≥30, <60	15	1 (6.7)	0 (0.0)
	1.25–5 mg^c^	≥60	36	1 (2.8)	1 (2.8)

Stratified by age					
Study name	Esaxerenone dose	Baseline age (years)	*N*	Serum potassium ≥5.5 mEq/L, *n* (%)	Serum potassium ≥6.0 or ≥5.5 mEq/L on two consecutive occasions, *n* (%)
J203 [131]/J301 [25]	Fixed dose^b^	<65	742	22 (3.0)	2 (0.3)
	1.25–5 mg	≥65	182	8 (4.4)	4 (2.2)
		≥75	24	3 (12.5)	1 (4.2)
J302 [27]	Incremental dose	<65	290	15 (5.2)	2 (0.7)
	2.5–5 mg^c^	≥65	78	5 (6.4)	2 (2.6)
		≥75	6	0 (0.0)	0 (0.0)
J305 (unpublished data)	Incremental dose	<65	17	1 (5.9)	0 (0.0)
	1.25–5 mg^c^	≥65	41	6 (14.6)	0 (0.0)
		≥75	16	3 (18.8)	0 (0.0)
J306 [26]	Incremental dose^a^	<65	27	1 (3.7)	0 (0.0)
	1.25–5 mg^c^	≥65	24	1 (4.2)	0 (0.0)
		≥75	7	0 (0.0)	0 (0.0)

Stratified by type 2 diabetes with albuminuria					
Study name	Esaxerenone dose	Type 2 diabetes with albuminuria	*N*	Serum potassium ≥5.5 mEq/L, *n* (%)	Serum potassium ≥6.0 or ≥5.5 mEq/L on two consecutive occasions, *n* (%)
J203 [131]/J301 [25]	Fixed dose^b^ 1.25–5 mg	No	924	30 (3.2)	6 (0.6)
J302 [27]	Incremental dose 2.5–5 mg^c^	No	368	20 (5.4)	4 (1.1)
J306 [26]	Incremental dose 1.25–5 mg^c^	Yes	51	2 (3.9)	1 (2.0)

Patients receiving esaxerenone in each study were included; patients receiving placebo and other drugs were excluded. J203 was a randomized, double-blind, placebo-controlled, phase II clinical study in patients with essential hypertension. J204 was a randomized, double-blind, placebo-controlled, phase II clinical study in patients with type 2 diabetes with microalbuminuria. The patient populations for the other studies are noted in earlier tables

*eGFR* estimated glomerular filtration rate

^a^Includes all patients with serum potassium elevation, whether or not elevated potassium was reported as a side effect

^b^Non-approved administration regimen

^c^Combination treatment with CCB or RAS inhibitor

## Management of hyperkalemia during antihypertensive treatment with esaxerenone

Clinical data from phase III studies showed that serum potassium levels returned to normal after withdrawal of esaxerenone, and no additional treatment was required. In the J301 and J302 studies, esaxerenone treatment was discontinued in five and four patients, respectively, with serum potassium measurements of ≥6.0 or ≥5.5 mEq/L on two consecutive occasions. In all patients, potassium levels normalized within 28 days [25, 27].

Available clinical study data can be used to develop a profile of patients at higher risk of developing hyperkalemia, allowing appropriate selection of patients for esaxerenone therapy. Based on patient factors such as lower renal function (reduced eGFR, albuminuria) and older age, gradually increasing the esaxerenone dosage from 1.25 to 2.5–5 mg/day has been suggested as a good strategy to reduce the risk of developing hyperkalemia compared with starting treatment at a fixed dosage [22].

An exposure–response analysis was performed using data from clinical studies where esaxerenone was given at a fixed dosage in patients with hypertension and diabetic nephropathy [126] or where the esaxerenone dosage was gradually increased in patients with moderate renal impairment or diabetic nephropathy (Daiichi Sankyo Co., Ltd., unpublished data, J305 study; and published data, J306 study [26]). The analysis showed that event rates of the first occurrence of hyperkalemia increased as esaxerenone mean exposure increased when the dosage was fixed but not when esaxerenone was initiated at a lower dosage with gradual upwards titration [129]. In fact, in hypertensive patients with type 2 diabetes and albuminuria, the proportions of patients with serum potassium ≥5.5 mEq/L and serum potassium ≥6.0 or 5.5 mEq/L on two or more consecutive occasions were lower when esaxerenone was started at 1.25 mg/day and then increased [26] than when therapy was started at a higher fixed dosage [126] (Table 4). These data indicate that incremental dosing of esaxerenone increased serum potassium to a lesser extent (i.e., up to 5.5 mEq/L and rarely increased to ≥6.0 or 5.5 mEq/L on two or more consecutive occasions) when compared with the fixed dosing method. Although similar decreases in the incidence rate of hyperkalemia were reported when eplerenone was administered starting at a lower dose that gradually increased [130], careful monitoring of serum potassium is required before initiation of and during any up- or down-titration regimen. In the recently published J306 study, esaxerenone was administered over 12 weeks at a starting dosage of 1.25 mg/day and was gradually titrated to 2.5 mg/day and 5 mg/day at weeks 4, 6, or 8 based mainly on serum potassium levels but with reference to other patient factors such as eGFR and blood pressure [26]. In this case, 1/51 patients had consecutive serum potassium measurements >5.5 mEq/L, but this elevated level resolved following dosage reduction, indicating that careful monitoring and treatment modulation can be helpful in minimizing hyperkalemic risk. Because serum potassium increases may occur more frequently in patients with moderate renal dysfunction, it is desirable to monitor the values in patients with diabetes and albuminuria or proteinuria, elderly people, and patients with concomitant medications that induce hyperkalemia. As previously stated, the package insert recommends that serum potassium levels be measured before treatment initiation and at regular intervals thereafter [22].

**Table 4:** Reduction of serum potassium elevation by gradually increasing doses^a^

	Incremental dose	Fixed dose^b^		
Esaxerenone dosage (mg/day)	Total 1.25–5	1.25	2.5	5
Other antihypertensive drugs	ARB or ACE inhibitor	ARB or ACE inhibitor		
Study name	J306 [26]	J204 [126]		
Total *N*	51	70	68	69
Number of patients with adverse events	25 (49.0)	48 (68.6)	46 (67.6)	44 (63.8)
Serum potassium ≥5.5 mEq/L	2 (3.9)	8 (11.4)	9 (13.2)	14 (20.3)
Serum potassium ≥6.0 or ≥5.5 mEq/L on two consecutive occasions	1 (2.0)	2 (2.9)	2 (2.9)	7 (10.1)

Data are shown as *n* (%)

*ARB* angiotensin II-receptor blockers, *ACE* angiotensin-converting enzyme

^a^Includes all patients with serum potassium elevation, whether or not elevated potassium was reported as a side effect

^b^Non-approved administration regimen

## Conclusions

MR blockers, including the new agent esaxerenone, have antihypertensive activity and organ protective effects. However, a careful approach to therapy and proper management of serum potassium is required to ensure safe clinical use of these agents. This includes awareness of at-risk patient groups, choosing appropriate dosages for therapy initiation and dosage titration, and monitoring of serum potassium during therapy. It is important that physicians take these important factors into consideration to optimize MR blocker therapy in patients with hypertension.

## Supplementary information

Supplementary Table 1

Supplementary Table 2

Supplementary Table 3

Supplementary Table 4
