# Supplementary material for: Management of hyperkalemia during treatment with mineralocorticoid receptor blockers: findings from esaxerenone
Source: Hypertens Res. 2020 Nov 20;44(4):371–85. doi: 10.1038/s41440-020-00569-y (PMC8019656; doi:10.1038/s41440-020-00569-y)
Supplement: Supplementary file 1 — Supplementary Table 1 [file 41440_2020_569_MOESM1_ESM.docx]

## Supplementary Table 1. Timing of onset of serum potassium elevation in the J305 study of combined administration of esaxerenone and RAS inhibitor (unpublished data), in patients with moderate renal impairment^1^

| **Day (week)** | **Total (esaxerenone 1.25**–**5 mg)**  ***N* = 58** | **Dose increased**  ***n* = 54** | **No dose increase (1.25 mg)  *n* = 4** |
| --- | --- | --- | --- |
| **Serum potassium levels ≥5.5 mEq/L** | | | |
| 8 (1) | 1 (1.7) | 0 (0.0) | 1 (25.0) |
| 15 (2) | 1 (1.7) | 0 (0.0) | 1 (25.0) |
| 40 (6) | 1 (1.7) | 1 (1.9) | 0 (0.0) |
| 43 (6) | 2 (3.4) | 2 (3.7) | 0 (0.0) |
| 46 (6) | 1 (1.7) | 1 (1.9) | 0 (0.0) |
| 85 (12) | 1 (1.7) | 1 (1.9) | 0 (0.0) |

Data are shown as *n* (%).

^1^ Includes all patients with serum potassium elevation, whether or not elevated potassium was reported as a side effect.

RAS, renin-angiotensin system
