# Supplementary material for: Management of hyperkalemia during treatment with mineralocorticoid receptor blockers: findings from esaxerenone
Source: Hypertens Res. 2020 Nov 20;44(4):371–85. doi: 10.1038/s41440-020-00569-y (PMC8019656; doi:10.1038/s41440-020-00569-y)
Supplement: Supplementary file 2 — Supplementary Table 2 [file 41440_2020_569_MOESM2_ESM.docx]

## Supplementary Table 2. Timing of onset of serum potassium elevation in the J204 study of combined administration of esaxerenone and RAS inhibitor,^126^ in patients with type 2 diabetes and albuminuria^1^

| **Week** | **Placebo**  ***N* = 70** | **Esaxerenone dose^2^** | | | | |
| --- | --- | --- | --- | --- | --- | --- |
|  |  | **0.625 mg *n* = 68** | **1.25 mg**  ***n* = 70** | **2.5 mg  *n* = 68** | **5 mg *n* = 69** | **All doses**  ***N* = 275** |
| **Serum potassium levels ≥5.5 mEq/L** | | | | | | |
| Day 4 | 0 (0.0) | 0 (0.0) | 0 (0.0) | 2 (2.9) | 0 (0.0) | 2 (0.7) |
| 1 | 0 (0.0) | 1 (1.5) | 1 (1.4) | 0 (0.0) | 1 (1.4) | 3 (1.1) |
| 2 | 0 (0.0) | 0 (0.0) | 0 (0.0) | 1 (1.5) | 3 (4.3) | 4 (1.5) |
| 3 | 2 (2.9) | 0 (0.0) | 0 (0.0) | 1 (1.5) | 2 (2.9) | 3 (1.1) |
| 4 | 0 (0.0) | 0 (0.0) | 3 (4.3) | 1 (1.5) | 4 (5.8) | 8 (2.9) |
| 6 | 0 (0.0) | 1 (1.5) | 1 (1.4) | 1 (1.5) | 0 (0.0) | 3 (1.1) |
| 8 | 0 (0.0) | 0 (0.0) | 0 (0.0) | 1 (1.5) | 1 (1.4) | 2 (0.7) |
| 10 | 0 (0.0) | 1 (1.5) | 1 (1.4) | 0 (0.0) | 2 (2.9) | 4 (1.5) |
| 11 | 0 (0.0) | 0 (0.0) | 0 (0.0) | 0 (0.0) | 1 (1.4) | 1 (0.4) |
| 12 | 0 (0.0) | 0 (0.0) | 2 (2.9) | 2 (2.9) | 1 (1.4) | 5 (1.8) |
| **Serum potassium levels ≥6.0 mEq/L or ≥5.5 mEq/L on two consecutive occasions** | | | | | | |
| Day4 | 0 (0.0) | 0 (0.0) | 0 (0.0) | 0 (0.0) | 0 (0.0) | 0 (0.0) |
| 1 | 0 (0.0) | 1 (1.5) | 0 (0.0) | 0 (0.0) | 0 (0.0) | 1 (0.4) |
| 2 | 0 (0.0) | 0 (0.0) | 0 (0.0) | 0 (0.0) | 1 (1.4) | 1 (0.4) |
| 3 | 0 (0.0) | 0 (0.0) | 0 (0.0) | 1 (1.5) | 0 (0.0) | 1 (0.4) |
| 4 | 0 (0.0) | 0 (0.0) | 0 (0.0) | 0 (0.0) | 3 (4.3) | 3 (1.1) |
| 6 | 0 (0.0) | 0 (0.0) | 1 (1.4) | 0 (0.0) | 1 (1.4) | 2 (0.7) |
| 8 | 0 (0.0) | 0 (0.0) | 0 (0.0) | 1 (1.5) | 0 (0.0) | 1 (0.4) |
| 10 | 0 (0.0) | 0 (0.0) | 0 (0.0) | 0 (0.0) | 1 (1.4) | 1 (0.4) |
| 11 | 0 (0.0) | 0 (0.0) | 0 (0.0) | 0 (0.0) | 1 (1.4) | 1 (0.4) |
| 12 | 0 (0.0) | 0 (0.0) | 1 (1.4) | 0 (0.0) | 0 (0.0) | 1 (0.4) |

Data are shown as *n* (%).

^1^ Includes all patients with serum potassium elevation, whether or not elevated potassium was reported as a side effect.

^2^ Non-approved administration regimen.

RAS, renin-angiotensin system
