# Supplementary material for: Management of hyperkalemia during treatment with mineralocorticoid receptor blockers: findings from esaxerenone
Source: Hypertens Res. 2020 Nov 20;44(4):371–85. doi: 10.1038/s41440-020-00569-y (PMC8019656; doi:10.1038/s41440-020-00569-y)
Supplement: Supplementary file 3 — Supplementary Table 3 [file 41440_2020_569_MOESM3_ESM.docx]

## Supplementary Table 3. Timing of onset of serum potassium elevation (J302 long-term administration study^27^)^1^

| **Week** | **Total**  **(2.5-5 mg)**  ***N* = 368** | **Esaxerenone monotherapy**  ***n* = 245** | **Combination with RAS inhibitor**  ***n* = 64** | **Combination with CCB**  ***n* = 59** |
| --- | --- | --- | --- | --- |
| **Serum potassium ≥5.5 mEq/L** | | | | |
| 2 | 3 (0.8) | 3 (1.2) | 0 (0.0) | 0 (0.0) |
| 4 | 1 (0.3) | 0 (0.0) | 1 (1.6) | 0 (0.0) |
| 6 | 3 (0.8) | 1 (0.4) | 1 (1.6) | 1 (1.7) |
| 8 | 3 (0.8) | 2 (0.8) | 1 (1.6) | 0 (0.0) |
| 10 | 2 (0.5) | 2 (0.8) | 0 (0.0) | 0 (0.0) |
| 12 | 0 (0.0) | 0 (0.0) | 0 (0.0) | 0 (0.0) |
| **2–12 total** | **12 (3.3)** | **8 (3.3)** | **3 (4.7)** | **1 (1.7)** |
| 16 | 3 (0.8) | 2 (0.8) | 1 (1.6) | 0 (0.0) |
| 20 | 2 (0.5) | 2 (0.8) | 0 (0.0) | 0 (0.0) |
| 24 | 0 (0.0) | 0 (0.0) | 0 (0.0) | 0 (0.0) |
| 28 | 1 (0.3) | 1 (0.4) | 0 (0.0) | 0 (0.0) |
| 32 | 1 (0.3) | 0 (0.0) | 0 (0.0) | 1 (1.7) |
| 36 | 1 (0.3) | 1 (0.4) | 0 (0.0) | 0 (0.0) |
| 40 | 0 (0.0) | 0 (0.0) | 0 (0.0) | 0 (0.0) |
| 44 | 0 (0.0) | 0 (0.0) | 0 (0.0) | 0 (0.0) |
| 48 | 0 (0.0) | 0 (0.0) | 0 (0.0) | 0 (0.0) |
| 52 | 0 (0.0) | 0 (0.0) | 0 (0.0) | 0 (0.0) |
| **Serum potassium levels ≥6.0 mEq/L or ≥5.5 mEq/L on two consecutive occasions** | | | | |
| 2 | 1 (0.3) | 1 (0.4) | 0 (0.0) | 0 (0.0) |
| 10 | 1 (0.3) | 1 (0.4) | 0 (0.0) | 0 (0.0) |
| **2–12 total** | **2 (0.5)** | **2 (0.8)** | **0 (0.0)** | **0 (0.0)** |
| 20 | 1 (0.3) | 1 (0.4) | 0 (0.0) | 0 (0.0) |
| 36 | 1 (0.3) | 1 (0.4) | 0 (0.0) | 0 (0.0) |

Data are shown as *n* (%).

^1^ Includes all patients with serum potassium elevation, whether or not elevated potassium was reported as a side effect.

CCB, calcium channel blocker; RAS, renin-angiotensin system.
