# Supplementary material for: Management of hyperkalemia during treatment with mineralocorticoid receptor blockers: findings from esaxerenone
Source: Hypertens Res. 2020 Nov 20;44(4):371–85. doi: 10.1038/s41440-020-00569-y (PMC8019656; doi:10.1038/s41440-020-00569-y)
Supplement: Supplementary file 4 — Supplementary Table 4 [file 41440_2020_569_MOESM4_ESM.docx]

## Supplementary Table 4. Incidence of serum potassium elevation by sex, body weight, hepatic function, and other hypertensive drugs

| **Stratified by sex** | | | | | |
| --- | --- | --- | --- | --- | --- |
| **Study name** | **Esaxerenone dose** | **Sex** | ***N*** | **Serum potassium ≥5.5 mEq/L,**  ***n* (%)^3^** | **Serum potassium ≥6.0 or ≥5.5 mEq/L on two consecutive occasions,**  ***n* (%)^3^** |
| J203^131^/J301^25^ | 1.25–5 mg^1^ | Male | 665 | 25 (3.8) | 3 (0.5) |
|  |  | Female | 259 | 5 (1.9) | 3 (1.2) |
| J302^27^ | 2.5–5 mg^2^ | Male | 286 | 18 (6.3) | 4 (1.4) |
|  |  | Female | 82 | 2 (2.4) | 0 (0.0) |
| **Stratified by body weight** | | | | | |
| **Study name** | **Esaxerenone dose** | **Baseline body weight (kg)** | ***N*** | **Serum potassium ≥5.5 mEq/L,**  ***n* (%)^3^** | **Serum potassium ≥6.0 or ≥5.5 mEq/L on two consecutive occasions,**  ***n* (%)^3^** |
| J203^131^/J301^25^ | 1.25–5 mg^1^ | < median (69.1 kg) | 465 | 14 (3.0) | 3 (0.6) |
|  |  | ≥ median | 459 | 16 (3.5) | 3 (0.7) |
| J302^27^ | 2.5–5 mg^2^ | < median (70.4 kg) | 184 | 11 (6.0) | 4 (2.2) |
|  |  | ≥ median | 184 | 9 (4.9) | 0 (0.0) |
| **Stratified by hepatic function** | | | | | |
| **Study name** | **Esaxerenone dose** | **Enzyme (U/L)** | ***N*** | **Serum potassium ≥5.5 mEq/L,**  ***n* (%)^3^** | **Serum potassium ≥6.0 or ≥5.5 mEq/L on two consecutive occasions,**  ***n* (%)^3^** |
| J203^131^/J301^25^ | 1.25–5 mg^1^ | **ALT** |  |  |  |
|  |  | <45 | 827 | 26 (3.1) | 6 (0.7) |
|  |  | ≥45 | 97 | 4 (4.1) | 0 (0.0) |
|  |  | **AST** |  |  |  |
|  |  | <40 | 864 | 28 (3.2) | 6 (0.7) |
|  |  | ≥40 | 60 | 2 (3.3) | 0 (0.0) |
| J302^27^ | 2.5–5 mg^2^ | **ALT** |  |  |  |
|  |  | <45 | 327 | 17 (5.2) | 4 (1.2) |
|  |  | ≥45 | 41 | 3 (7.3) | 0 (0.0) |
|  |  | **AST** |  |  |  |
|  |  | <40 | 340 | 19 (5.6) | 4 (1.2) |
|  |  | ≥40 | 28 | 1 (3.6) | 0 (0.0) |
| **Stratified by other antihypertensive drugs** | | | | | |
| **Study name** | **Esaxerenone dose** | **Treatment combination** | ***N*** | **Blood potassium increased^4^**  ***n* (%)** | - |
| J302^27^ | 2.5–5 mg^2^ | No | 253 | 11 (4.3) | - |
|  |  | RAS inhibitor | 18 | 0 (0.0) | - |
|  |  | CCB | 93 | 3 (3.2) | - |

^1^ Non-approved administration regimen.

^2^ Combination treatment with CCB or RAS inhibitor.

^3^ Includes all patients with serum potassium elevation, whether or not elevated potassium was reported as a side effect.

^4^ Blood potassium increased was reported as an adverse event based on MedDRA/J V20.0.

ALT, alanine aminotransferase; AST, aspartate aminotransferase; CCB, calcium channel blocker; RAS, renin-angiotensin system.
